# Clinical signs and utility of CT PET scan in eosinophilic fasciitis?

**DOI:** 10.1002/ski2.439

**Published:** 2024-12-01

**Authors:** Sarah R. Adamson, John C. Su, Sally Ng, Christopher Fong

**Affiliations:** ^1^ Department of Dermatology Eastern Health Box Hill Victoria Australia; ^2^ Eastern Health Clinical School Monash University Melbourne Victoria Australia; ^3^ Epworth Eastern Epworth Health Box Hill Victoria Australia; ^4^ Department of Plastic & Reconstructive Surgery Eastern Health Box Hill Victoria Australia; ^5^ Department of Rheumatology Eastern Health Box Hill Victoria Australia

## Abstract

A 61 year old male presented with clinical signs of Eosinophilic fasciitis (EF), a rare connective tissue disease. Early recognition of the diagnosis of EF is essential. Common examination findings are prayer sign and distal limb swelling, induration, venous guttering, and peau d'orange. CT PET scan can be helpful in supporting the diagnosis of EF.

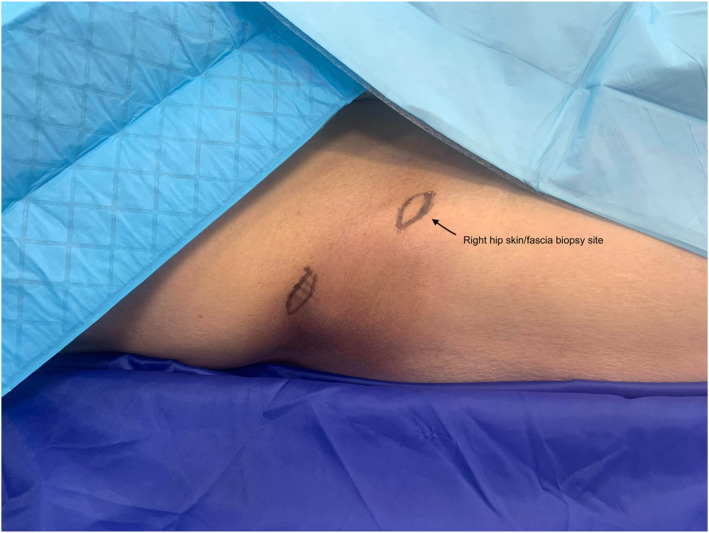

A 61‐year‐old Chinese Australian male presented 4 months after the acute onset of a large, red, itchy, indurated patch over his hip (Figure 1), followed by acute, itchy, inflammatory leg swelling, then forearm swelling several weeks later. He had experienced 4 kg weight loss, night sweats, difficulty climbing stairs and opening jars. He had been reviewed by several specialists who were treating him for cardiac failure and lymphoedema prior to his presentation to the rheumatology department and then to the dermatology department.

**FIGURE 1:**
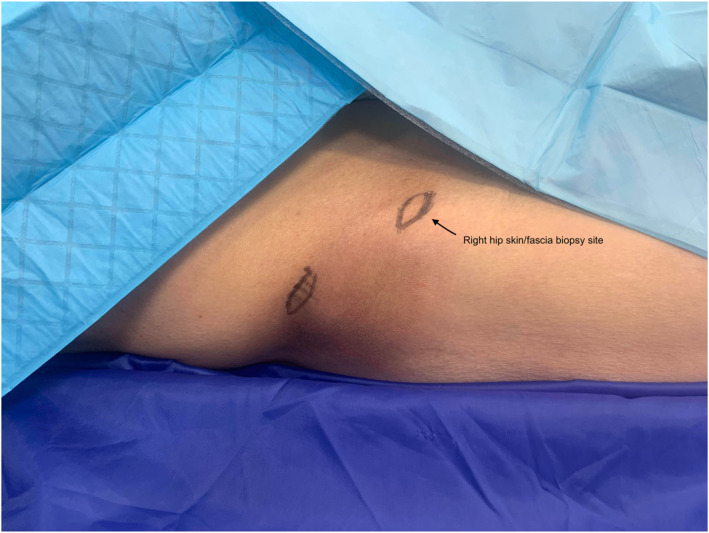
Right thigh biopsy site over area of induration.

His past medical history included type 2 diabetes mellitus, hypertension and dyslipidaemia. His regular medications included metformin, linagliptin, perindopril and atorvastatin, of which the latter two he had commenced 2 months prior.

On examination, he had distal limb induration and hyperpigmentation, with venous guttering, exacerbated by elevation (Figure 2). Prayer sign was present, with inability to oppose more than just the fingertips, and limited wrist extension (Figure 3). Lower limbs were oedematous, shiny, hyperpigmented and indurated with associated peau d'orange (Figure 4). He had no palpable lymphadenopathy. His fingertips and nails were normal.

**FIGURE 2:**
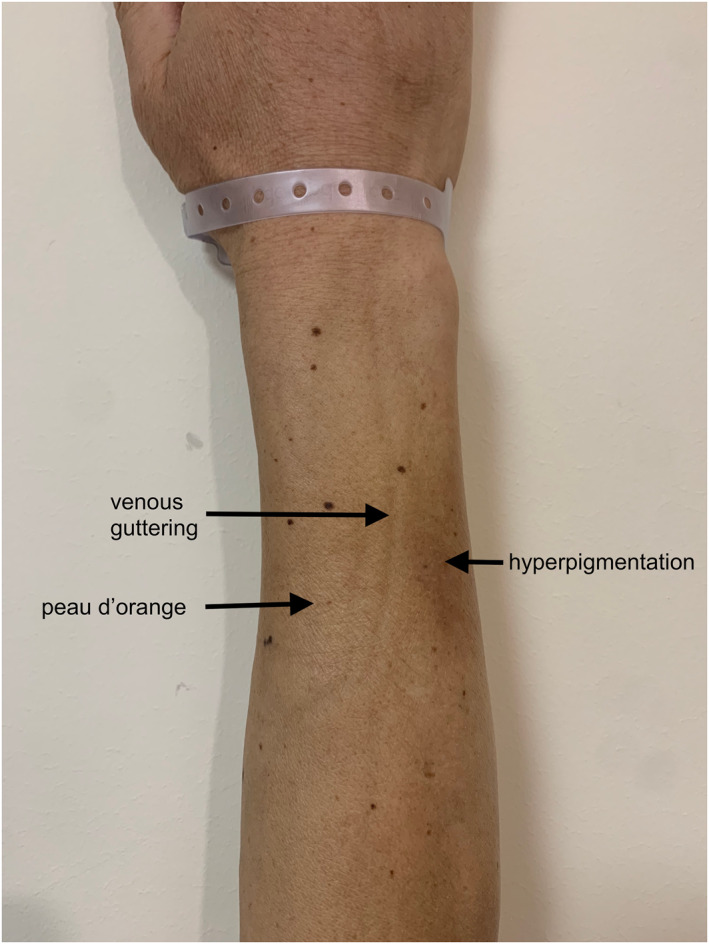
Right forearm hyperpigmentation, venous guttering and peau d'orange.

**FIGURE 3:**
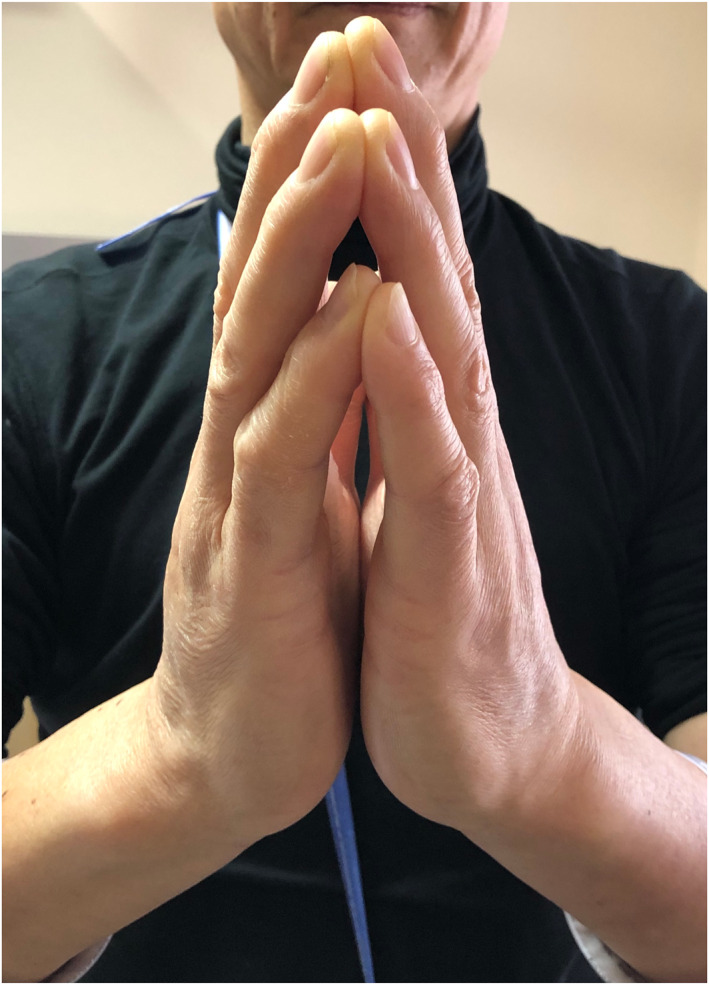
Prayer sign.

**FIGURE 4:**
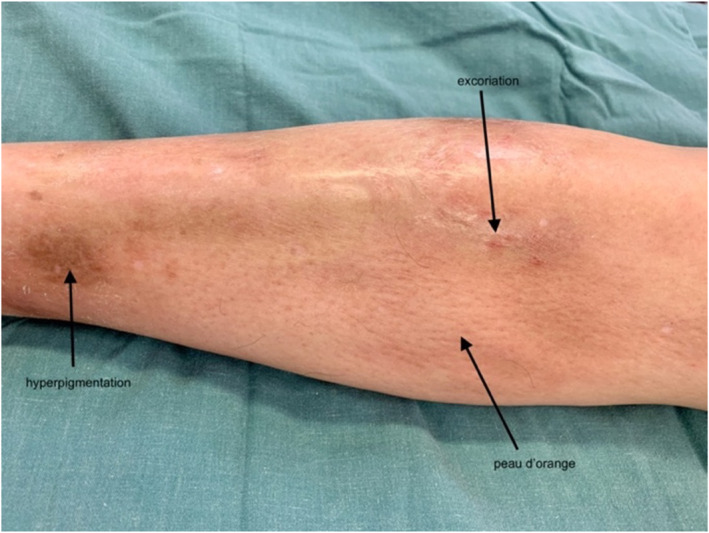
Right leg oedematous, shiny, hyperpigmented and excoriated with peau d'orange present.

ESR was elevated (40 mm/hr), but blood count, renal and liver function were normal. PET CT scan showed extensive increased fascial uptake (Figure 5a–c). PET CT was performed rather than an MRI as it was more helpful and time‐efficient to delineate the extent of fascial involvement, and to exclude an underlying malignancy as the patient also had a suspicious lung nodule. An incisional skin and fascial biopsy showed dermal and subcutaneous sclerodermoid histology.

**FIGURE 5:**
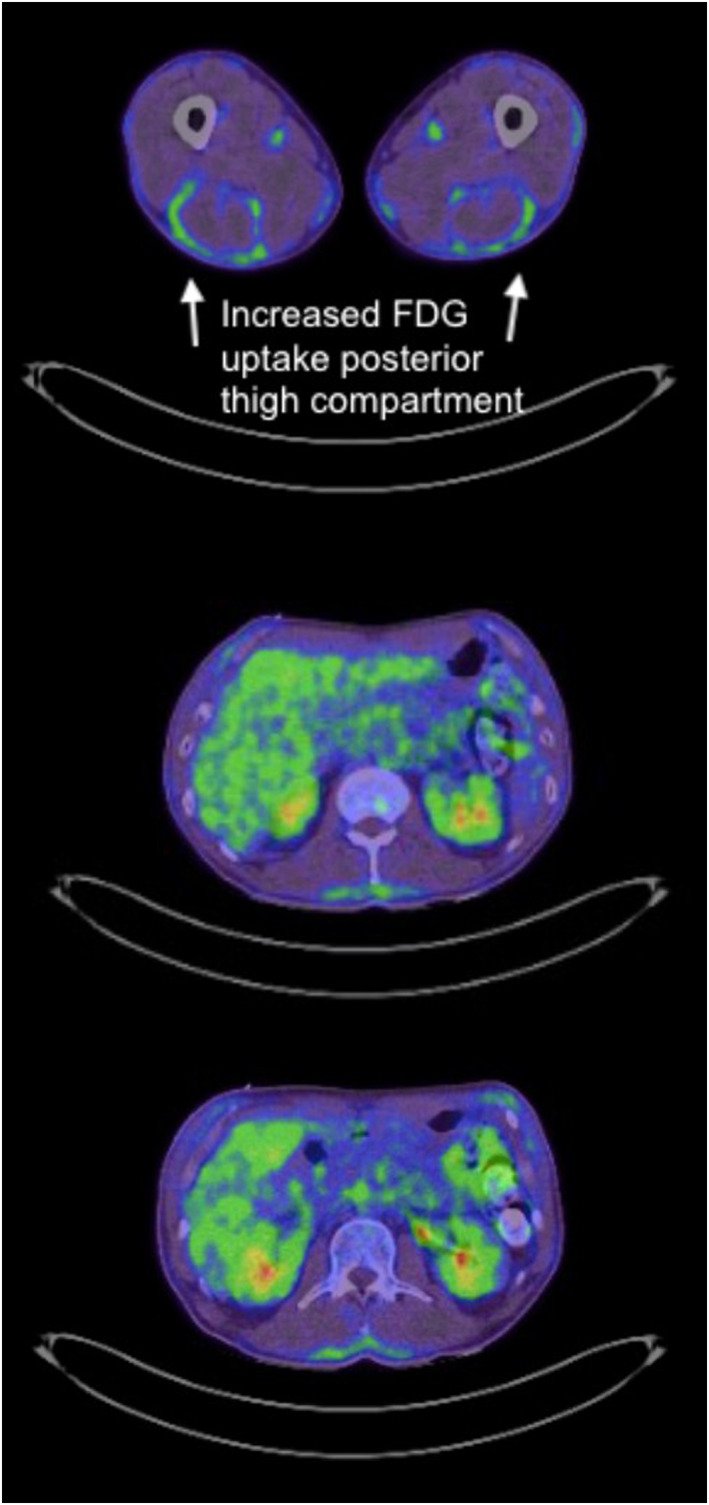
(a–c) PET scan of thighs and trunk showing diffuse increased fascial FDG uptake.

The most likely diagnosis was eosinophilic fasciitis (EF). The differentials were scleroderma; however, the patient had fingertip sparing, and ANA was negative. The primary concern was to exclude an underlying malignancy, particularly that of bone marrow.

EF is a rare connective tissue disease whose incidence, aetiology and pathogenesis are uncertain.^1^ Strenuous exercise and certain medications such as atorvastatin,^2^ radiation therapy, infections and paraneoplastic syndromes have been identified as triggers. Generally, 70%–83% of patients present with symmetrical upper and lower limb signs.^2^ Investigations may show eosinophilia, hypergammaglobulinaemia and elevated inflammatory markers such as ESR.^2^ There was no peripheral eosinophilia in this case; however, this patient presented late, and this is not a requirement for diagnosis. Full thickness skin biopsy including fascia is the gold standard for diagnosis.^2^ MRI is often performed to support the diagnosis of EF,^2^ and CT PET scans may also be of value^3^, ^4^ showing increased fascial uptake and extent of fascial involvement (Figure 5). It is important to exclude an underlying malignant aetiology, particularly a haematological malignancy.^5^

The patient was started on oral prednisolone initially at 50 mg daily and mycophenolate titrating up to 1.5 g twice daily. Over the past 12 months his skin has improved.

## CONFLICT OF INTEREST STATEMENT

None to declare.

## AUTHOR CONTRIBUTIONS

**Sarah R. Adamson**: Conceptualization (equal); data curation (equal); investigation (equal); methodology (equal); resources (equal); validation (equal); writing – original draft (lead); writing – review & editing (lead). **John C. Su**: Conceptualization (equal); data curation (equal); formal analysis (equal); investigation (lead); methodology (equal); validation (equal); writing – original draft (equal); writing – review & editing (equal). **Sally Ng**: Conceptualization (equal); writing – original draft (supporting); writing – review & editing (supporting). **Christopher Fong**: Conceptualization (supporting); data curation (supporting); investigation (equal); writing – original draft (supporting); writing – review & editing (supporting).

## ETHICS STATEMENT

Approval from Epworth Healthcare, Office of Research obtained (Reference Number: EH2024‐1160).

## PATIENT CONSENT

Written patient consent was obtained for publication.

## Data Availability

The data underlying this article will be shared with the corresponding author on reasonable request.
